# Genetic diversity in the endangered terrestrial orchid *Cypripedium japonicum* in East Asia: Insights into population history and implications for conservation

**DOI:** 10.1038/s41598-018-24912-z

**Published:** 2018-04-24

**Authors:** Huai Zhen Tian, Li Xia Han, Jun Li Zhang, Xing Lin Li, Takayuki Kawahara, Tomohisa Yukawa, Jordi López-Pujol, Pankaj Kumar, Myong Gi Chung, Mi Yoon Chung

**Affiliations:** 1 School of Life Sciences, East China Normal University, Shanghai, 200241 China; 2 Hokkaido Research Center, Forestry and Forest Products Research Institute, Sapporo, Hokkaido, Japan; 3 Tsukuba Botanical Garden, National Museum of Nature and Science, Tsukuba, Ibaraki, Japan; 4 Botanic Institute of de Barcelona (IBB, CSIC-ICUB), Passeig del Migdia s/n, Barcelona, 08038 Spain; 5 Kadoorie Farm & Botanic Garden, Lam Kam Rd., Lam Tsuen, Tai Po, New Territories, Hong Kong SAR, China; 6 Division of Life Science and the Research Institute of Natural Science (RINS), Gyeongsang National University, Jinju, 52828 Republic of Korea; 7 RINS, Gyeongsang National University, Jinju, 52828 Republic of Korea

## Abstract

Little is known about levels and patterns of genetic diversity for the entire range of endangered orchids native to China, Korea, and Japan. In this study, we focus on *Cypripedium japonicum* and suggest three hypotheses: 1) that genetic drift has been a primary evolutionary force; 2) that populations in central and western China harbor higher levels of genetic variation relative to those from eastern China; and 3) that *C*. *japonicum* in China maintains the highest genetic variation among the three countries. Using ISSR and SCoT markers, we investigated genetic diversity in 17 populations to test the three hypotheses. As anticipated, we found low levels of genetic diversity at the species level with substantially high degree of genetic divergence, which can be mainly attributed to random genetic drift. Chinese populations harbor the highest within-population genetic variation, which tends to increase from east to west. We also found a close relationship between Korean populations and central/western Chinese populations. Historical rarity coupled with limited gene flow seems to be important factors for shaping genetic diversity and structure of *C*. *japonicum*. Our results indicate that the mountain areas in central and western China were likely refugia at the Last Glacial Maximum.

## Introduction

The lady’s slipper orchid genus *Cypripedium* L., comprising ca. 50 species of terrestrial herbs, is distributed throughout subtropical to temperate regions of the Northern Hemisphere with the exception of northern Africa^1^. Although East Asia and North America represent the two major centers of diversity for this genus, the former region harbors the majority of diversity with 32 species^2^. *Cypripedium japonicum* Thunb. belongs to section *Flabellinervia*^2^ and is distributed across East Asia in China, Korea, and Japan^3^. In China, it is the only slipper orchid that is distributed from western to eastern regions. Possessing two large fan-shaped leaves and highly appealing flowers, *C*. *japonicum* is of high horticultural value. In addition, its roots and rhizomes, and even the whole plant, are used in Traditional Chinese Medicine (TCM) to promote blood circulation for regulating menstruation, to dispel “wind” (in TCM, wind is one of the six “excesses” or external cases of disease) and to alleviate pain^4–6^. Although *C*. *japonicum* is widely distributed in East Asia, the size and number of populations have recently declined because of the destruction of their habitats^2,5,7^. It is found only on humus-rich soil under mature and successional deciduous forests on mountainous slopes partly as a result of narrow mycorrhizal specificity^8^. Although the species is not listed as threatened in the Chinese Red List, it will be included in the second batch of “National Key Protected Wild Plants” as a “Class I protected species”^9^. In Japan, it is categorized as “Vulnerable” (VU)^10^, whereas in Korea, where it is known to be extremely rare, is listed as “Critically Endangered” (CR)^11^. At the global level, *C*. *japonicum* is listed as “Endangered” (EN)^5^ and in need of protection and conservation. Under this situation, sound long-term scientific conservation strategies for protecting and conserving the wild populations of *C*. *japonicum* are badly needed; such strategies should be based on thorough knowledge of the species’ biology, especially its genetic diversity and structuring within the whole distribution area^12^.

Population genetic theory predicts that small size and spatial isolation of populations will lead to the random fixation of alleles (i.e., random genetic drift, RGD), promoting allele frequency divergence and inbreeding as well as a reduction in heterozygosity within populations. Overall, RGD reduces genetic diversity within populations and enhances genetic differentiation between populations. Chung *et al*.^7^ tried to test this prediction in six Korean populations of *C*. *japonicum* but, unfortunately, found no allozyme-based genetic diversity within and among populations. Qian *et al*.^13^, using amplified fragment length polymorphisms (AFLP), investigated six populations of *C*. *japonicum* in Tianmu Mts. of Zhejiang Province (eastern China) and found low polymorphism (21.0%) and high genetic differentiation (*G*_ST_ = 0.518). Qian *et al*.^4^, using inter-simple sequence repeats (ISSR), also found extremely low levels of within-population genetic variation (*H*_eP_ = 0.042) and substantially high among-population genetic differentiation (*G*_ST_ = 0.671) in six populations from eastern and central China. Biological and ecological traits such as small population size and founder effects, coupled with limited pollen and seed dispersal across isolated fragmented landscapes, have been suggested as factors for shaping genetic diversity and structure of *C*. *japonicum*^4,7^. According to population genetic theory, RGD should affect levels and the distribution of genetic diversity within and among populations, but with a relatively little effect on the level of genetic diversity within the species as a whole. If RGD has operated in populations of *C*. *japonicum* as a major evolutionary force for a long period of time, we further expect extremely low levels of genetic variation at the population level but low to moderate levels of genetic diversity at the species level, and substantially high genetic divergence between populations recovered from the full geographic range of this orchid.

The current geographical distribution of plant taxa is assumed to be the result of both present and historical factors (e.g., Pleistocene climate oscillations), being the latter important drivers in shaping the genetic structure and phylogeographical patterns of plants^14,15^. High levels of genetic diversity are expected for those areas that are thought to be refugial^16–21^ (but see^22^), as plants would have endured the most adverse periods maintaining relatively large populations, and also because populations having persisted in refugia would have had a longer demographic history than those that are the result of post-glacial colonization. Mountains anywhere played a key role as refugia for biota during the Neogene and Quaternary global cooling^23–25^; reasons include their relative eco-environmental stability and the need of mountain species for moving considerably shorter distances to track climatic oscillations as compared with lowland species^21^.

**Figure 1:**
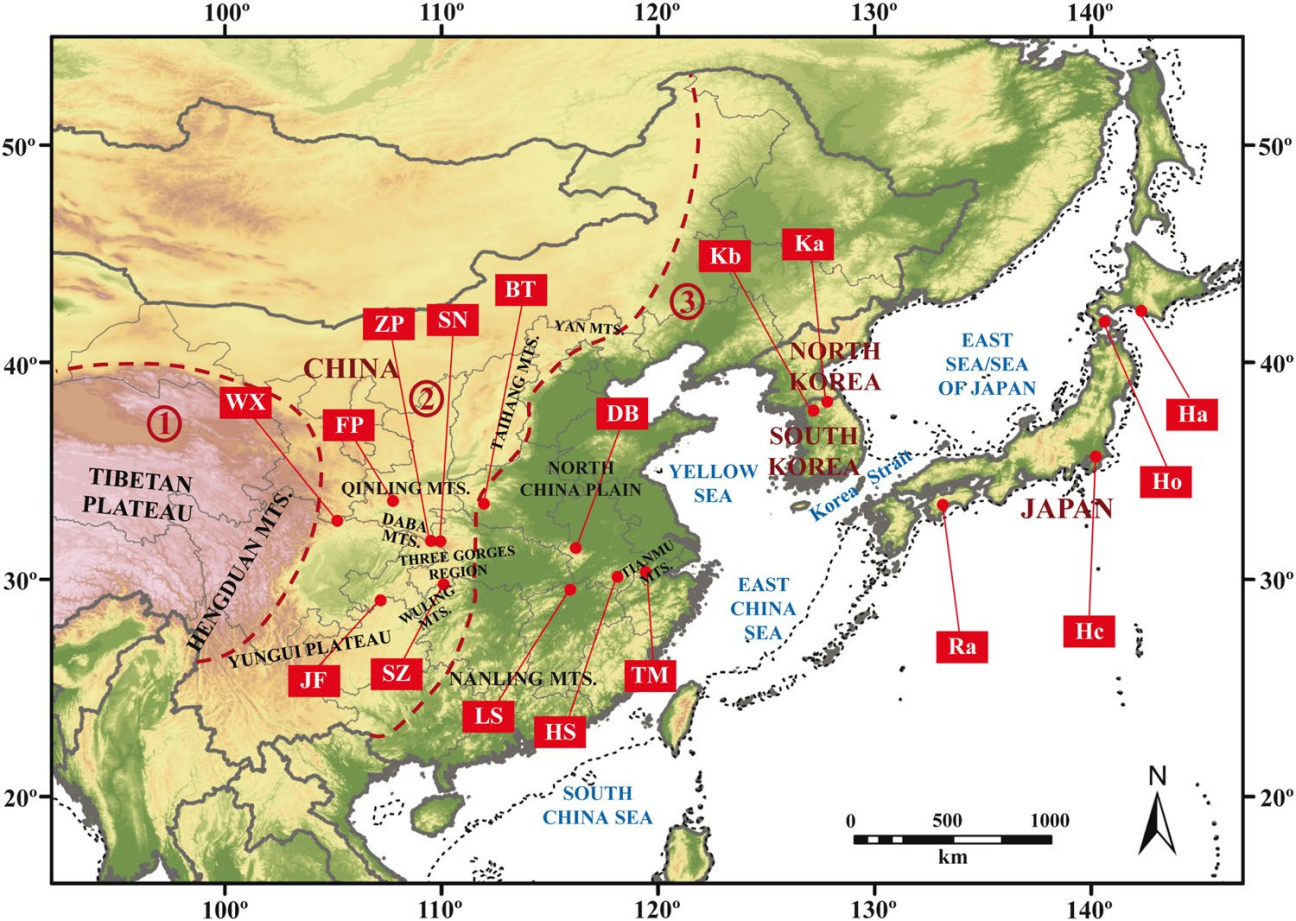
Sampled populations of *Cypripedium japonicum* in this study. The reconstructed Last Glacial Maximum coastlines are represented as thin dotted lines. All the geographical features quoted in the text are also indicated. The areas indicated by ①, ②, and ③ partitioned by thick dotted lines represent the “first”, the “second”, and “third” steps of the “three-step ladder” of Chinese geographic features^58^. The base map has been generated with ArcGIS 9.3 (ESRI, Redlands, CA, USA) from a 30 arc-sec layer downloaded from WorldClim Version 1 (http://worldclim.org/), and modified using Adobe Illustrator CS5.1 (Adobe Systems Incorporated, San Jose, CA, USA). Layers from WorldClim Version 1 are licensed under a Creative Commons Attribution-ShareAlike 4.0 International License (https://creativecommons.org/licenses/by-sa/4.0/).

Our study area in East Asia, the so-called “Sino-Japanese Floristic region” was not glaciated during the Last Glacial Maximum (LGM, ca. 21,000 yr before present) except for a few high-elevation mountains^21,26^. In China, several mountainous areas served as important East Asian Pleistocene refugia at the LGM for boreal and temperate species; such areas are mainly located in the western and the central parts of the country (e.g., the Hengduan Mts. and the central China Mountains, including the Qinling Mts., Daba Mts., and the Three Gorges Mountain Region; Fig. 1) but not exclusively (e.g., the Nanling Mts., Fig. 1; see also Fig. 3 in López-Pujol *et al*.^25^ and Fig. 4 in Qiu *et al*.^26^). If this scenario is also true for *C*. *japonicum* in China, we expect relatively higher levels of genetic variation in western populations compared to eastern ones. In addition, we can also anticipate that Chinese populations would harbor higher within-population genetic variation than Japanese and Korean ones, given that the main East Asian Quaternary refugia were located in China^26^; Chinese mountains usually reach higher altitudes (having, thus, larger altitudinal gradients), which allowed plants to track glacial-interglacial climate oscillations by means of vertical shifts^16,17^.

Despite several previous population genetic studies of *C*. *japonicum* based on small regional scale sampling have generated considerable background population genetics knowledge of this species^4,7,13^, a comprehensive study based on a large number of populations across its whole range in East Asia is necessary for interpreting the dynamics of variation over the range of the species, which would allow to design more efficient conservation strategies at both regional and global scales. Therefore, a thorough understanding of population genetic diversity and structuring of *C*. *japonicum* across its full range is desirable.

The genetic variation and relationships among wild populations can be efficiently determined by using different molecular markers^27^, and they can be combined to reach more comprehensive conclusions. Neutral ISSR markers have been widely used for detection of DNA polymorphism in rare species^28^ due to their advantage of being fast, simple, and cost effective, with good reproducibility^4,29^. In addition, the start codon targeted (SCoT) polymorphisms, which were developed^30^ based on the translation start codon, are simple, highly polymorphic, and abundant in the genome^27^. As the SCoT loci are gene-based markers and are likely to be subject to selection (i.e., non-neutral)^31^, their use in population genetic studies may be compromised. However, many studies have shown that they are highly correlated with genuinely neutral markers such as ISSR, and are usually more informative and effective than the latter^32–34^.

Using ISSR and SCoT markers, in the present study we estimated the levels and distribution of genetic diversity of *C*. *japonicum* sampled from China, Korea, and Japan to test the three hypotheses presented above (i.e., low levels of genetic variation and high genetic divergence between populations for this orchid, higher levels of genetic variation in western Chinese populations compared to eastern ones, and higher levels of genetic variation in Chinese populations compared to Japanese and Korean ones). With the resulting genetic information, along with available demographic information, we tried to provide recommendations for efficient *in situ* conservation and *ex situ* preservation of this rare and endangered orchid.

## Results

### Genetic Diversity

With ISSR, a total of 78 bands were scored with an average of 7.8 bands per primer, and 45 polymorphic bands were obtained. At the population level, the mean *PPB*_P_ was 9.7%, ranging from 1.3% (BT, LS) to 24.4% (FP), whereas the mean *N*_aP_, *PA*, *N*_eP_, *H*_EP_, and *SI* were 1.10, 1.88, 1.04, 0.027, and 0.042, respectively (see the Methods section or Table 1 for the definitions of abbreviations of genetic parameters). Population FP exhibited the highest *H*_EP_ value (0.062), while population BT did the lowest *H*_EP_ (0.004). At the species level, all these parameters exhibited higher mean values, with *PPB*_S_ of 57.7%, *N*_aS_ of 1.58, *N*_eS_ of 1.16, *H*_ES_ of 0.104, and *SI* of 0.169 (Table 1). With SCoT, a total of 77 bands were observed with an average of 8.6 bands per primer, of which 47 were polymorphic. At the population level, the mean *PPB*_P_ was 10.9%, ranging from 2.6% (Ka) to 24.7% (FP), and the mean *N*_aP_, *PA*, *N*_eP_, *H*_EP_, and *SI* were 1.11, 1.69, 1.05, 0.033, and 0.051, respectively (Table 1). FP exhibited the highest *H*_EP_ value (0.074) again, but Ka had the lowest *H*_EP_ (0.007). At the species level, like the ISSR, all those parameters exhibited higher mean values: *PPB*_S_ = 61.0%, *N*_aS_ = 1.61, *N*_eS_ = 1.24, *H*_ES_ = 0.145, and *SI* = 0.224 (Table 1).

**Table 1:** Population information and summary of levels of genetic diversity of *Cypripedium japonicum* based on ISSR/SCoT analysis.

Country	Province or Prefecture	Altitude (m)	*n*		*PPB*(%)		*N*_a_ [*PA*]		*N*_e_		*H*_E_ (SD)		*SI* (SD)	
Region			ISSR	SCoT	ISSR	SCoT	ISSR	SCoT	ISSR	SCoT	ISSR	SCoT	ISSR	SCoT
Population														
**CHINA**														
*Eastern*														
DB (Dabieshan)	Anhui	200	5	5	7.7	7.8	1.08 [3]	1.08 [3]	1.05	1.05	0.031 (0.110)	0.029 (0.103)	0.045 (0.159)	0.044 (0.152)
HS (Huangshan)	Anhui	1000	12	12	7.7	9.1	1.08 [5]	1.09 [0]	1.02	1.07	0.016 (0.066)	0.040 (0.128)	0.027 (0.103)	0.057 (0.182)
LS (Lushan)	Jiangxi	1200	3	3	1.3	3.9	1.01 [1]	1.04 [3]	1.01	1.03	0.006 (0.050)	0.017 (0.087)	0.008 (0.072)	0.025 (0.124)
TM (Tianmushan)	Zhejiang	1100	27	27	11.5	13.0	1.12 [1]	1.13 [5]	1.02	1.04	0.017 (0.052)	0.027 (0.091)	0.032 (0.093)	0.044 (0.137)
**Mean**					**7**.**1**	**8**.**5**	**1**.**07 [2**.**50]**	**1**.**09 [2**.**75]**	**1**.**03**	**1**.**05**	**0**.**018 (0**.**010)**	**0**.**028 (0**.**009)**	**0**.**028 (0**.**015)**	**0**.**043 (0**.**013)**
*Central*														
BT (Baotianman)	Henan	1000	21	21	1.3	5.2	1.01 [0]	1.05 [0]	1.01	1.03	0.004 (0.035)	0.017 (0.075)	0.006 (0.055)	0.026 (0.114)
SN (Shennongjia)	Hubei	1160	22	23	19.2	18.2	1.19 [5]	1.18 [4]	1.06	1.06	0.039 (0.101)	0.041 (0.101)	0.066 (0.156)	0.068 (0.158)
**Mean**					**10**.**3**	**11**.**7**	**1**.**10 [2**.**50]**	**1**.**12 [2**.**00]**	**1**.**03**	**1**.**05**	**0**.**022 (0**.**025)**	**0**.**029 (0**.**017)**	**0**.**036 (0**.**042)**	**0**.**047 (0**.**029)**
*Western*														
FP (Foping)	Shaanxi	1260	21	21	24.4	24.7	1.24 [3]	1.24 [1]	1.10	1.12	0.062 (0.132)	0.074 (0.147)	0.098 (0.197)	0.115 (0.219)
JF (Jinfo)	Chongqing	1000	8	8	15.4	18.2	1.15 [6]	1.18 [3]	1.08	1.12	0.050 (0.125)	0.071 (0.158)	0.077 (0.187)	0.104 (0.228)
SZ (Sangzhi)	Hunan	1000	21	21	20.5	23.4	1.21 [1]	1.23 [4]	1.09	1.08	0.059 (0.128)	0.054 (0.113)	0.092 (0.194)	0.089 (0.177)
WX (Wenxian)	Gansu	1200	20	20	12.8	13.0	1.13 [2]	1.13 [2]	1.05	1.05	0.034 (0.104)	0.035 (0.100)	0.053 (0.155)	0.055 (0.154)
ZP (Zhenping)	Shaanxi	1230	20	na	12.8	na	1.13 [1]	na [na]	1.06	na	0.039 (0.113)	na	0.060 (0.168)	na
**Mean**					**17**.**2**	**19**.**8**	**1**.**17 [2**.**60]**	**1**.**20 [2**.**50]**	**1**.**08**	**1**.**09**	**0**.**049 (0**.**012)**	**0**.**058 (0**.**018)**	**0**.**076 (0**.**020)**	**0**.**091 (0**.**026)**
**Mean in China**					**12**.**2**	**13**.**7**	**1**.**12 [2**.**55]**	**1**.**14 [2**.**50]**	**1**.**05**	**1**.**07**	**0**.**031 (0**.**020)**	**0**.**040 (0**.**020)**	**0**.**051 (0**.**030)**	**0**.**063 (0**.**031)**
**KOREA**														
Ka (Hwacheon)	Gangwon	590	23	23	1.6	2.6	1.03 [0]	1.03 [0]	1.01	1.01	0.008 (0.051)	0.007 (0.046)	0.012 (0.077)	0.012 (0.074)
Kb (Pocheon)	Gyeonggi	520	20	18	5.1	5.2	1.05 [1]	1.05 [0]	1.01	1.04	0.007 (0.034)	0.022 (0.096)	0.013 (0.061)	0.032 (0.139)
**Mean in Korea**					**3**.**4**	**3**.**9**	**1**.**04 [0**.**50]**	**1**.**04 [0**.**00]**	**1**.**01**	**1**.**03**	**0**.**008 (0**.**000)**	**0**.**015 (0**.**010)**	**0**.**013 (0**.**001)**	**0**.**022 (0**.**014)**
**JAPAN**														
Ha (Niikappu)	Hokkaido	50	7	7	3.9	3.9	1.04 [2]	1.04 [0]	1.02	1.03	0.013 (0.067)	0.015 (0.077)	0.019 (0.100)	0.022 (0.112)
Hc (Yotsukaido)	Chiba	30	19	19	7.7	9.1	1.08 [0]	1.09 [0]	1.04	1.03	0.025 (0.093)	0.021 (0.070)	0.038 (0.139)	0.035 (0.115)
Ho (Hokuto)	Hokkaido	70	14	15	7.7	9.1	1.08 [0]	1.09 [2]	1.05	1.05	0.029 (0.106)	0.031 (0.108)	0.042 (0.153)	0.046 (0.157)
Ra (Nakatosa)	Kochi	400	22	22	5.1	7.8	1.05 [1]	1.08 [0]	1.03	1.05	0.017 (0.082)	0.030 (0.111)	0.025 (0.118)	0.044 (0.159)
**Mean in Japan**					**6**.**1**	**7**.**5**	**1**.**06 [0**.**75]**	**1**.**08 [0**.**50]**	**1**.**04**	**1**.**04**	**0**.**021 (0**.**007)**	**0**.**024 (0**.**008)**	**0**.**031 (0**.**011)**	**0**.**037 (0**.**011)**
**Mean (total)**			16.8	15.6	**9**.**7**	**10**.**9**	**1**.**10 [1**.**88]**	**1**.**11 [1**.**69]**	**1**.**04**	**1**.**05**	**0**.**027 (0**.**018)**	**0**.**033 (0**.**019)**	**0**.**042 (0**.**029)**	**0**.**051 (0**.**030)**
**Species level**			**285**	**265**	**57**.**7**	**61**.**0**	**1**.**58**	**1**.**61**	**1**.**16**	**1**.**24**	**0**.**104 (0**.**155)**	**0**.**145 (0**.**188)**	**0**.**169 (0**.**227)**	**0**.**224 (0**.**266)**

Abbreviations: *n*, sample size; *PPB* (%), percentage of polymorphic bands; *N*_a_, observed number of alleles; *PA*, number of private alleles; *N*_e_, average effective number of alleles per locus; *H*_E_, Nei’s gene diversity index; SD, standard deviation; *SI*, Shannon’s information index; na, not available.

Mean genetic estimates (mean *PPB*_P_, *N*_aP_, *N*_eP_, *H*_EP_, and *SI*) from SCoT were higher than those from ISSR (Table 1), and these differences were significant (Wilcoxon signed-rank tests; for all genetic parameters *P* < 0.05; see Supplementary Table S1). However, we found a significant correlation between ISSR and SCoT datasets (Spearman’s rank correlation test; for all genetic parameters *R*^2^, coefficient of determination, ranged from 0.791 for *H*_EP_ to 0.930 for *PPB*_P_ and *N*_aP_, *P* < 0.000; Supplementary Table S1), suggesting these two datasets are highly compatible and, thus, that SCoT markers behaved as selectively (or nearly selectively) neutral in *C*. *japonicum*. For *PA*, however, we found neither significant difference nor correlation between the two datasets (Supplementary Table S1).

In China, levels of genetic diversity with the exception of *PA* and *N*_eP_ tended to increase from east to west (Table 1); populations in western region harbored significantly higher levels of within-population genetic variation than those in eastern region (with ISSR, mean *H*_EP_ = 0.049 vs. 0.018, *P* = 0.008; with SCoT, mean *H*_EP_ = 0.058 vs. 0.028, *P* = 0.030). On the other hand, levels of genetic diversity of the six parameters tended to increase from Korea to China (Table 1); 11 Chinese populations exhibited marginally significantly higher levels of within-population genetic variation than two Korean populations (with ISSR, mean *H*_EP_ = 0.031 vs. 0.008, *P* = 0.100; with SCoT, mean *H*_EP_ = 0.040 vs. 0.015, *P* = 0.054).

### Genetic differentiation between populations

Values of *G*_ST_ showed very small differences between the total set of populations and the set of populations excluding those with small sample sizes (*n* < 10), and this was applicable for the whole species range (e.g., for ISSR, *G*_ST_ = 0.749 and 0.737 including and excluding small populations, respectively) as well as within each country separately (e.g., for ISSR in China, *G*_ST_ = 0.636 vs. 0.599) and with all possible combinations of country pairs (e.g., for ISSR in China vs. Japan, 0.335 vs. 0.362). We consider, therefore, that including populations with small sample size would not significantly alter results and conclusions, and we conducted further data analyses with the total set of populations.

With ISSR, the total genetic diversity for the species (*H*_T_) was 0.106, while the mean heterozygosity within populations (*H*_S_) and between populations (*D*_S_) were 0.027 and 0.080, respectively. The degree of genetic differentiation between populations (*G*_ST_) was 0.749. Similar results were found with the SCoT dataset: *H*_T_ = 0.152, *H*_S_ = 0.033, *D*_S_ = 0.119, and *G*_ST_ = 0.779. Chinese populations showed higher *G*_ST_ (0.636 for ISSR and 0.697 for SCoT) than those from Japan (0.321 for ISSR and 0.406 for SCoT) and Korea (0.104 for ISSR and 0.145 for SCoT) (Supplementary Table S2). When populations in each country were combined, the highest *G*_ST_ value was found for “Korea vs. Japan” (0.672 for ISSR; 0.608 for SCoT), followed by “China vs. Japan” (0.335 for ISSR; 0.300 for SCoT), and “China vs. Korea” (0.120 for ISSR; 0.159 for SCoT) (Supplementary Table S2).

AMOVA analysis with ISSR revealed that variation among regions (China, Japan, and Korea) and among populations within regions contributed 48.1% and 30.9% to the total genetic variance, respectively (Table 2). Similar results were found with SCoT: 43.5% and 38.3% of the total variation among regions and among populations within regions, respectively (Table 2). Consistent with the hierarchical analyses for *G*_ST_, separate AMOVA analyses for different subsets of the data showed that the highest genetic divergence between regions was found between Korea and Japan (80.2% for ISSR and 75.4% for SCoT), followed by China vs. Japan (52.6% for ISSR and 43.3% for SCoT; Table 2); in contrast, low degree of divergence was found between China and Korea (22.6% for ISSR and 32.2% for SCoT; Table 2).

**Table 2:** Analysis of molecular variance (AMOVA) of *Cypripedium japonicum* based on ISSR/SCoT.

Source of variation	df		SS		VC		Variation (%)	
	ISSR	SCoT	ISSR	SCoT	ISSR	SCoT	ISSR	SCoT
Among three regions (China, Japan, Korea)	2	2	459.693	574.246	2.590	3.171	48.10**	43.49**
Among populations within regions	14	13	391.138	594.405	1.661	2.796	30.85**	38.34**
Within populations	268	249	303.723	329.938	1.133	1.325	21.05**	18.17**
Between two regions (China, Korea)	1	1	106.784	216.327	0.923	2.241	22.64**	32.18**
Among populations within regions	11	10	367.66	548.472	1.953	3.316	47.92**	47.61**
Within populations	210	190	251.878	267.458	1.199	1.408	29.43**	20.21**
Between two regions (China, Japan)	1	1	351.987	375.605	3.443	3.505	52.55**	43.25**
Among populations within regions	13	12	389.746	592.265	1.824	3.083	27.84**	38.04**
Within populations	227	210	291.627	318.322	1.285	1.516	19.61**	18.71**
Between two regions (Korea, Japan)	1	1	209.703	234.052	3.984	4.425	80.18**	75.39**
Among populations within regions	4	4	24.871	48.074	0.339	0.688	6.82**	11.73**
Within populations	99	98	63.94	74.095	0.646	0.756	13.00**	12.88**
Between two regions (China + Korea, Japan)	1	1	352.909	357.919	3.262	3.047	51.44**	38.76**
Among populations within regions	15	14	497.923	810.733	1.945	3.489	30.68**	44.38**
Within populations	268	249	303.723	329.938	1.133	1.325	17.87**	16.86**

Abbreviations: df, degrees of freedom; SS, sum of squares; VC, variance components.

**P < 0.01.

We found a significant correlation between pairwise genetic distances and linear geographic distances (km) of populations (Mantel test for IBD, isolation by distance: *r* = 0.713, *P* = 0.001 for ISSR; *r* = 0.515, *P* = 0.001 for SCoT) when all populations were used (Supplementary Fig. S1). Except for SCoT in Japan (*r* = 0.478, *P* = 0.262, primarily due to low statistical power, only six pairs), results of the hierarchical test for IBD showed significant correlations within countries (China, *r* = 0.752 for ISSR and *r* = 0.669 for SCoT; Japan, *r* = 0.858 for ISSR), and also significant correlations were observed when countries were combined (China vs. Japan, *r* = 0.782 for ISSR and *r* = 0.600 for SCoT; Korea vs. Japan, *r* = 0.687 for ISSR and *r* = 0.747 for SCoT; China vs. Korea, *r* = 0.316 for ISSR and *r* = 0.362 for SCoT) (Supplementary Table S2).

### Genetic structure and relationships between three countries

**Figure 2:**
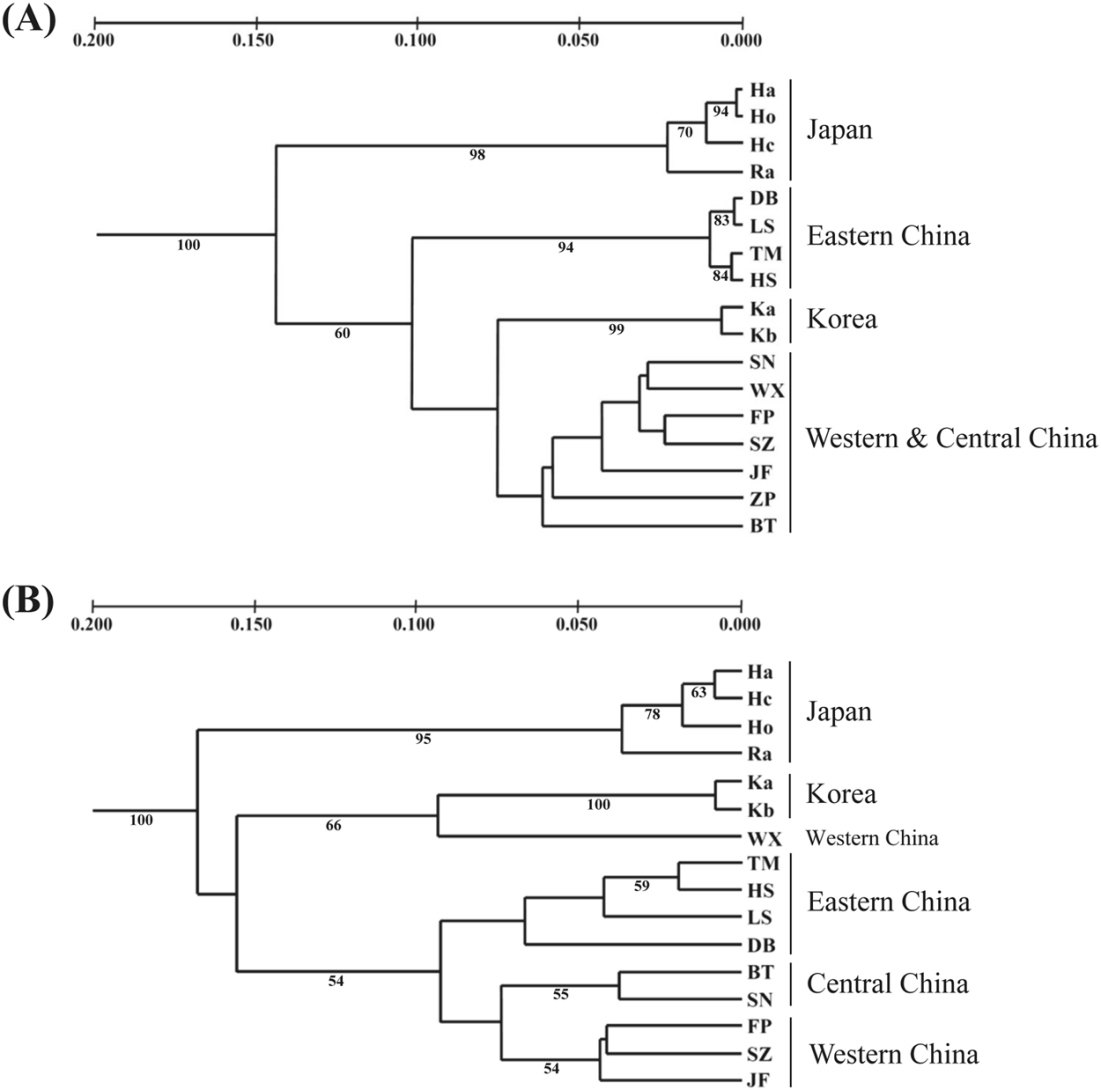
UPGMA dendrograms showing the relationships between the populations with ISSR (**A**) and SCoT (**B**). Numbers below branches represent bootstrap support (BS) for 999 replicates; only BS values ≥ 50% are provided.

The UPGMA dendrograms constructed from ISSR (Fig. 2A) and SCoT (Fig. 2B) markers both showed that the populations of *C*. *japonicum* could be largely divided into two geographic clusters: the China + Korea cluster and the Japanese cluster. The main difference between the two datasets was that Korean populations clustered with western and central Chinese populations with ISSR (even when deleting population ZP; data not shown), whereas they clustered only with WX (a western Chinese population) and then with the remaining Chinese populations with SCoT.

**Figure 3:**
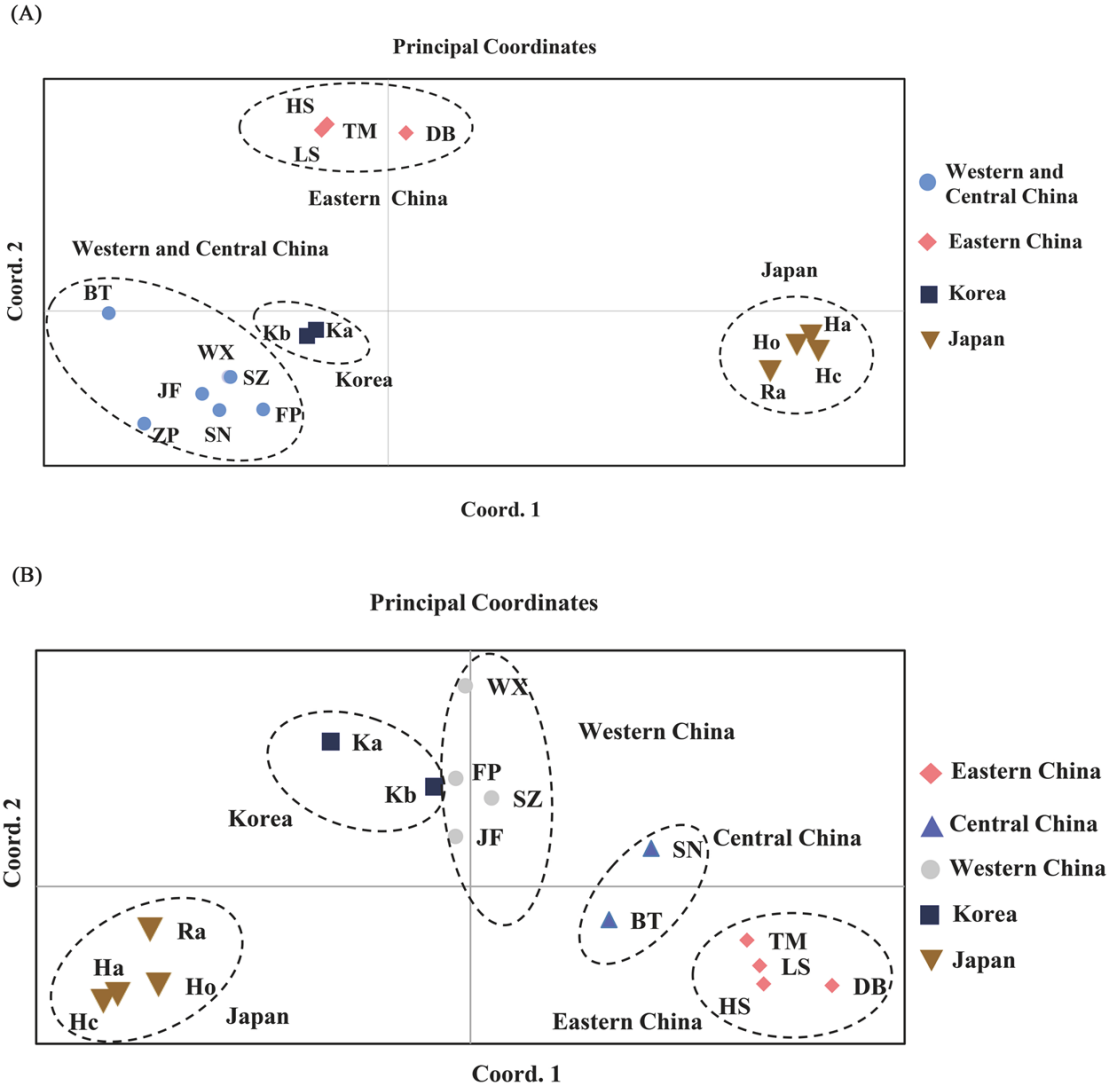
The principal coordinate analysis (PCoA) plots based on the two principal axes of ISSR (**A**) and SCoT (**B**) analysis.

Principal coordinate analysis (PCoA) results were in good agreement with those from the UPGMA dendrograms (Fig. 3). In the ISSR PCoA (Fig. 3A), the first two components accounted for 76.5% (axis 1 = 47.2%; axis 2 = 29.3%) of the total genetic variance, in a similar way that in the SCoT PCoA (Fig. 3B) in which they accounted for 70.8% (axis 1 = 44.7%; axis 2 = 26.1%) of the total genetic variance. The main differences between the two distribution plots were BT and SN populations, which were placed between the eastern and western Chinese populations in the SCoT analysis but nested within western Chinese populations in the ISSR analysis.

**Figure 4:**
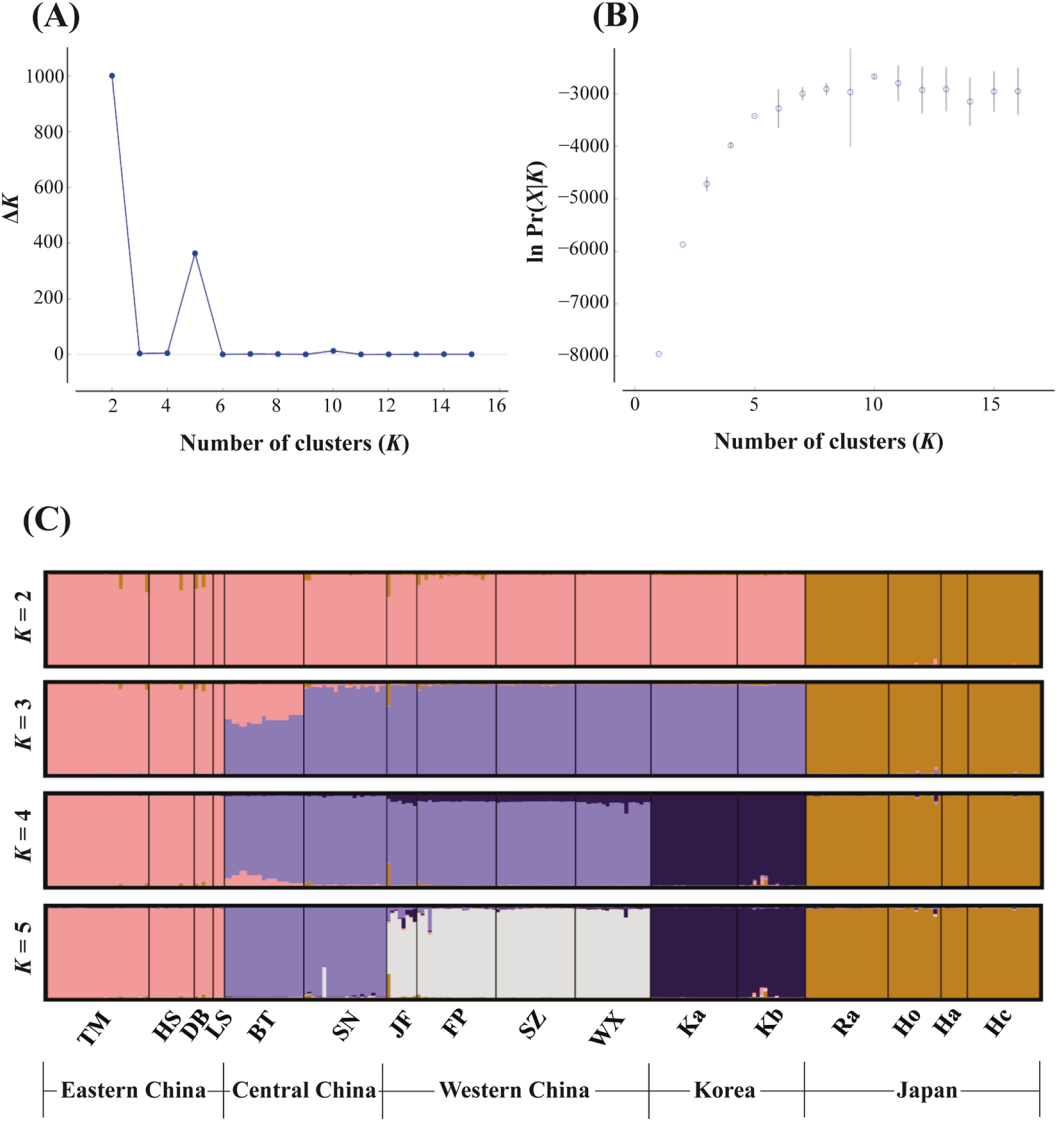
The most likely *K* was estimated from the Δ*K* statistics^76^ (**A**) and the log probability of data [ln Pr(*X*|*K*)] values^79^ (**B**) using Structure Harvester^77^, and Bayesian clustering analysis based on the combined ISSR and SCoT markers for 16 populations (ZP excluded) when *K* = 2 to *K* = 5 (**C**).

In the combined datasets (ISSR and SCoT), the Δ*K* statistics indicated *K* = 2 as the best grouping scheme (*K* = 5 was the second highest value; Fig. 4A), whereas the estimated log probability of data obtained with Structure reached a plateau when the number of clusters was set to 5 (Fig. 4B). Thus, we chose *K* = 5 as the most meaningful clustering. However, we also represented *K* = 2, *K* = 3, and *K* = 4 as these results might be informative. When *K* = 2, the Chinese and Korean populations clustered together and Japanese population had an independent genetic pool (Fig. 4C). However, when *K* = 5, the Japanese, Korean, eastern Chinese, western Chinese, and central Chinese populations represented independent genetic pools (Fig. 4C). Interestingly, at *K* = 3 the Korean populations were clustered together with central and western Chinese populations, and their own cluster were not assigned until *K* = 4 (Fig. 4C). The findings of Bayesian clustering analysis implemented in Structure were in broad agreement with the UPGMA and PCoA results, clearly depicting that 1) populations from China and Korea have a close relationship, and 2) Korean populations are genetically more related to central and western Chinese populations than to eastern Chinese populations.

## Discussion

### Comparisons between ISSR and SCoT

In this study, two dominant genetic markers, ISSR and SCoT, were employed to assess the levels and distribution of genetic diversity of *C*. *japonicum* in East Asia. The amplification products generated from the SCoT markers may be correlated to functional genes and their corresponding traits, while the ISSR markers detect polymorphisms in microsatellite regions that do not necessarily represent functional genes^35^. Each marker system targets different regions of the genome, which leads to different sources of the detected diversity. In the present study, both of them indicated low genetic diversity both at population and species levels, and showed a similar clustering of *C*. *japonicum* populations (see below for details). Although the values for the genetic parameters were significantly higher for SCoT compared to ISSR, overall a significant correlation was found between these two datasets. To sum up, using independent data from two different methods (as complementary analyses) can represent a more efficient and accurate way to survey the genetic variability of a given species.

### Extremely low levels of within-population and high degree of between population genetic diversity

As expected, *C*. *japonicum* exhibits extremely low levels of within-population genetic variation (with ISSR, *PPB*_P_ = 9.7% vs. 46.6%; *H*_EP_ = 0.027 vs. 0.167) when compared with other orchids that have been screened with ISSR (Supplementary Table S3). At the species level, *C*. *japonicum* still harbors low levels of genetic variation (with ISSR, *PPB*_S_ = 57.7% vs. 63.4%; *H*_ES_ = 0.104 vs. 0.263) compared to means found in orchids (Supplementary Table S3). The much greater estimates at the species level are due to the substantial genetic divergence detected between populations of *C*. *japonicum* (*G*_ST_ = 0.749 for ISSR; *G*_ST_ = 0.779 for SCoT). We found considerably high *G*_ST_ values within China (*G*_ST_ = 0.636 for ISSR; *G*_ST_ = 0.697 for SCoT) and Japan (*G*_ST_ = 0.321 for ISSR; *G*_ST_ = 0.406 for SCoT), which are likely to be caused primarily by RGD (see below) and limited gene flow. A very similar *G*_ST_ estimate was reported in the epiphytic or lithophytic orchid *Dendrobium fimbriatum* from Xishuangbanna (a region of ca. 19,700 km^2^ in Yunnan Province, South China); Ma and Yin^36^, using 12 ISSR markers, estimated high species-level genetic variation (*PPB*_S_ = 89.7%; *H*_ES_ = 0.323) and high degree of population differentiation *G*_ST_ (0.744) from five populations, and, accordingly, low population-level variation (*PPB*_P_ = 23.9%; *H*_EP_ = 0.087). We believe that, like *C*. *japonicum*, RGD coupled with restricted pollen and seed dispersal would have shaped the genetic structure of *D*. *fimbriatum* in Xishuangbanna.

Chung *et al*.^7^ found no allozyme variation in six Korean populations of *C*. *japonicum* and argued that the species was historically rare compared to its congener *C*. *macranthos* on the Korean Peninsula, suggesting that RGD would be a major evolutionary force in the former. Consistent with this and more recently, Son and Son^37^ found low levels of microsatellite-based genetic variation in four Korean populations of *C*. *japonicum* (*%P*_P_ = 35.0%; *A*_P_ = 1.53; *H*_eP_ = 0.109, based on 10 polymorphic loci) but a higher among-population divergence (*Φ*_ST_ = 0.810 including one Japanese population). Our finding of much lower levels of within-population genetic variation when compared to those at the species level, indirectly indicates the importance of RGD for *C*. *japonicum*. A recent meta-analysis of allozyme-based population genetic studies in orchids also revealed that “rare” taxa harbor significantly lower levels of genetic diversity than their “common” congeners (at the population level, percentage of polymorphic loci, *%P*_P_ = 46.7% vs. 23.6%; *H*_EP_ = 0.154 vs. 0.065 and at the species level, *%P*_S_ = 63.8% vs. 30.4%; *H*_ES_ = 0.190 vs. 0.072)^38^. One of the exceptions was *C*. *macranthos* var. *rebunense*, endemic to the small Rebun Island close to Hokkaido (Japan), which harbors comparable levels of genetic diversity with its common North American congeners. This unexpected genetic result is probably due to the fact that the decline in habitat range and population size of this island’s endemic variety has been very recent (since 1950s)^39^; there have not been sufficient generations for the initial diversity to be substantially eroded by RGD^40^.

Other life-history or ecological traits associated with pollen and seed dispersal have a highly significant impact on genetic variability and are strong predictors for the levels and distribution of genetic diversity in plant species^41,42^. The three bumblebees *Bombus remotus*, *B*. *imitator* and *B*. *picipes* are the most effective primary pollinators of *C*. *japonicum* in China^43^, while *B*. *ardens* and *B*. *diversus* are the effective pollinators in Japan^44^. It is known that bumblebees have a flight range of less than 15 km^45^. Pollen dispersal is, therefore, likely to be relatively restricted among the highly isolated populations of *C*. *japonicum*. Moreover, non-rewarding *C*. *japonicum* flowers bloom in May^43^, when precipitation is high in most of its distribution range in China, which could dramatically reduce the foraging activity of bumblebees and indirectly limit pollen dispersal^46^. Pollination also suffers from visits by illegitimate visitors as these insects destroy the flowers^47^. All these factors might contribute to the low pollen dispersal observed in the species^43,44^.

Since orchids produce tiny, “dust-like” numerous seeds, they have the potential for long-distance seed movement once seeds are released and enter the air column^48^. However, direct and indirect (genetic) studies have documented that the majority of seeds are transported to very short distances, in the close vicinity of maternal plants, with events of long-distance dispersal being sporadic^49–51^. In addition, like other terrestrial orchids, the seeds of *C*. *japonicum* require mycorrhizal fungi for germination and seedling nutrition^8,52,53^. The limitation of pollen and seed flow across highly isolated populations would lead to low gene flow, increasing the genetic differentiation between populations of *C*. *japonicum* (see below for further details).

### Levels of genetic diversity and population genetic structure: insights into phylogeographic history

Since historical events leave an “indelible” mark on patterns of genetic diversity found within plant species, understanding their current levels and distribution can provide valuable insights into the recent evolutionary and biogeographical history of these species^16,17,19,26,54^. As hypothesized, levels of genetic diversity (with the exception of *PA* and *N*_eP_) tend to increase from east to west in China. The finding that the central and western populations harbor relatively higher levels of genetic variation compared to eastern ones are consistent with the hypothesis that the former areas served as large LGM refugia for many species, mainly boreal and temperate plants, in China^25,26^. In particular, population FP, the highest in levels of genetic diversity among all Chinese populations examined, is located in the south Qinling Mts. (Fig. 1); this mountain region is considered one of the main refugia of western China, and was an area not heavily influenced by the LGM^55^. In general, large parts of central and southwestern China, including the Three Gorges Region, the Yungui Plateau, the Qinling Mts., and partially the Hengduan Mts. (Fig. 1), acted as plant refugia^25,26^ due to their relatively stable climatic conditions^56^, which allowed the persistence of even thermophilous, relict plant taxa (e.g., *Davidia involucrata*^57^). All the studied populations of *C*. *japonicum* occurring in these areas (with the only exception of population BT) are among the most genetically variable (Table 1). It should be noted that, although in eastern China the presence of LGM refugia has also been hypothesized, these were generally small (“microrefugia”), such as Tianmu Mts. (Fig. 1)^25,26^. Despite the levels of genetic variability for the eastern Chinese populations are smaller, their similar number of private alleles (*PA*) compared to western ones (Table 1) also suggests the occurrence of glacial refugia, albeit small, there.

The genetic clustering patterns of Chinese populations might, indeed, reflect the different nature of the terrain, which would have deeply influenced the occurrence of *C*. *japonicum* refugial areas. The two main genetic clusters detected in the species (the western/central China cluster and the eastern China cluster, clearly depicted by the UPGMA, PCoA, and Structure) roughly correspond to the “second” and “third” steps of the “three-step ladder” of Chinese geographic features^58^, respectively (see ② and ③ in Fig. 1, respectively). The second step approximately extends from the eastern margin of the Qinghai-Tibetan Plateau to Taihang Mts. (Fig. 1), and it is comprised of plateaus and middle-altitude mountains (of generally 1,000–2,000 m), including the Qinling Mts. and other mountain systems of central China (Daba Mts. and the Three Gorges area; Fig. 1), which would have served as the main glacial refugia for *C*. *japonicum* and many other species^25,26^; the third step, containing China’s largest plains, would have harbored, instead, populations that endured the LGM in small, favorable pockets in eastern China (in “microrefugia”, e.g., Tianmu Mts.; Fig. 1), and perhaps also newly-founded populations (i.e., populations of post-glacial origin).

As also hypothesized, the levels of genetic diversity of *C*. *japonicum* are higher for Chinese populations, followed by Japanese populations, and finally Korean ones, which further supports the species’ glacial refugial scenario for central and western China. Korean populations exhibit extremely low levels of genetic diversity, despite that the Baekdudaegan (BDDG, the main mountain system of the Korean Peninsula) was a glacial refugium for a large assemblage of boreal and temperate plants during the LGM^21,59^. Plants from the BDDG often harbor moderate to high levels of within-population genetic diversity (Table 1 in Chung *et al*.^21^), because these mountains might provide relatively stable habitats and enabled plants to track the climate changes by altitudinal movements, ensuring relatively large population sizes. For example, *C*. *macranthos* (a congener of *C*. *japonicum*), largely occurring along the BDDG ridge, harbors moderate levels of within-population genetic variation^7^. Populations of *C*. *japonicum*, however, occur on hillsides with elevations of less than 600 m on the Korean Peninsula. Likewise, in Japan, *C*. *japonicum* largely occurs on lower hillsides, and our samples were collected from hillsides at altitudes below 400 m. Like in Korea, lowlands and low-elevation hills in Japan hardly harbored large refugia (“macrorefugia”). Instead, contemporary populations would be descendants of populations that endured the LGM in microrefugia, where they suffered the effects of RGD.

The closer relationships between Korean and Chinese populations than between Korean and Japanese populations can be simply due to the fact that the Korean Peninsula is contemporarily connected with NE China, although such connection was even more straightforward at the LGM, when the Yellow Sea was completely exposed (*cf*. dotted lines for the reconstructed LGM coastlines; Fig. 1). The Japanese Archipelago, instead, was nearly isolated from the continent throughout the Quaternary. Unexpectedly, the two Korean populations are clustered with western and central Chinese populations rather than with the eastern Chinese populations (Figs 2–4), which are geographically closer to the Korean populations. An origin of the Korean populations from the mountains of western China instead of eastern China could explain such pattern of genetic clustering; since *C*. *japonicum* occurs in middle-low altitude mountains in China, the most straightforward migration routes were probably through the mountains of north China [Qinling, Taihang Mts., then the north Hebei Mts. (Yan Mts.); see Fig. 1]. Colonization of Korea from eastern Chinese mountains seems unlikely, as both regions are separated by large plains (e.g., the huge North China Plain; Fig. 1) with no suitable habitat for the species. Our ongoing study on phylogeography of *C*. *japonicum* in East Asia using cpDNA has revealed that the only haplotype found in Korean populations also occur in SN and ZP, which are located in Daba Mts. (L.X. Han *et al*. unpubl. data).

The results of AMOVA, UPGMA, PCoA, and Structure (but also the hierarchical analyses of *G*_ST_ and IBD) show that *C*. *japonicum* in East Asia can largely be grouped into two genetic clusters, namely the China + Korea cluster and the Japan cluster. The different chromosome number of *C*. *japonicum* in China (2*n* = 22 in Tianmu Mts.^60^) and Japan (2*n* = 20 based on cultivated plants^61,62^) suggests an ancient separation and a reproductive barrier between Chinese and Japanese populations, in case that these counts are confirmed for other populations within the species’ range in these two areas. Our genetic data that indicate a sharp genetic break between continental (China + Korea) and Japanese populations are in agreement with the cytogenetic divergence. Whether both genetic lineages (China + Korea and Japan) represent cryptic species, and merit any taxonomic (as species, subspecies or varieties) or evolutionary recognition (e.g., evolutionarily significant units), need further research including morphological, cytogenetic (e.g., its chromosome number in Korea is not known), and molecular phylogenetic studies.

### Implications for conservation

*Cypripedium japonicum* is threatened with extinction because of its low levels of genetic variation and its high ornamental and medicinal value, particularly in China. The species is categorized as “Endangered” at global scale following the most recent criteria of IUCN^63^: B2ab(ii,iii,iv,v); C2a(i)^5^. Thus, *in situ* and *ex situ* conservation efforts should be of particular importance for this species. Some previously recorded populations in China might have disappeared already due to deforestation and/or illegal collection^4,5^.

For a short-time conservation strategy, *C*. *japonicum* should be considered as the priority subject of *in situ* conservation with an emphasis on maintaining the number of genetically distinct individuals. Designation of priority protected areas for natural habitats of *C*. *japonicum* could provide ideal protection for *in situ* conservation^64^. In Korea, fortunately, all known Korean populations are protected by laws^11^.

For a long-term conservation strategy, the four populations in central and western China with highest genetic diversity (FP, SZ, SN and JF) should be prioritized for protection, because such populations would provide the best source of propagules for *ex situ* conservation and *in situ* restoration efforts^65^. Although populations from Japan exhibit low within-population genetic variation, they should also be considered for *in situ* conservation and *ex situ* preservation because of their considerable divergence from the Chinese plus Korean populations. Indeed, the extinction of any population may lead to a considerable loss of genetic diversity because of the species’ high degree of between-population genetic differentiation, restricted distribution pattern and narrow habitat preferences. Thus, all populations are of great importance for short-term and long-term survival of *C*. *japonicum* in East Asia.

Since about 20 conservation actions have already been recommended for Korean, Chinese, and overall populations in East Asia^4,5,7^, we here further provide a few suggestions for the management and conservation of *C*. *japonicum*: 1) to use hand-pollination within or among populations in order to enhance the fruit set and even to accelerate the rejuvenation of small populations because of low rate of fruit set (5.2–7.7% in Hubei, China^43^; 14.9% in Chiba, Japan^44^); 2) to apply tissue culture techniques for propagation of different genotypes as well as for satisfying the demand of traditional medicine; 3) to identify detailed clonal structure and fine-scale genetic structure of typical or representative populations in the three countries where the species is present (China, Korea, Japan), in order to provide more scientific and efficient sampling for *ex situ* preservation; and 4) to develop comprehensive conservation strategies through international collaboration among concerned countries.

### Conclusions

To our knowledge, this study is the first detailed examination of any East Asian orchid covering its entire distribution range to evaluate the levels of genetic diversity, to provide hypotheses about the past phylogeographic history, and to recommend conservation strategies. As expected, the endangered terrestrial orchid *C*. *japonicum* harbors extremely low genetic diversity at the population level across its whole distribution area. At the species level (samples as a whole), *C*. *japonicum* maintains low levels of genetic diversity with high degree of genetic divergence between populations. These genetic results suggest that random genetic drift (RGD) coupled with limited gene flow between populations have played a significant role in shaping levels of genetic diversity and structure in the species. Genetic data revealed that the populations are genetically arranged into two major groups: Chinese + Korean and Japanese groups. Although *in situ* conservation and *ex situ* preservation strategies should be primarily focused on populations with high genetic diversity in central and western China, conservation of Korean and Japanese populations is also desirable through close international cooperation among concerned countries.

## Methods

### Study species

*Cypripedium japonicum* grows in moist humus-rich soil in the understory of mature and successional deciduous forests on hillsides from China, Japan, and Korea. The plants can also propagate via buds that grow from the creeping underground rhizomes^2^. Most plants of *C*. *japonicum* have two leaves and a 35–55 cm tall stalk bearing a single, large, pendent flower with sac-like labellum (4–5 × 3–3.5 cm). *Cypripedium japonicum* is self-compatible with a non-rewarding, deceptive pollination system and requires effective pollinators^43,44,47^. Five species of the genus *Bombus* have been reported as effective pollinators both in China and Japan with a low fruit set of 5.2–14.9%^43,44^. Seeds of *C*. *japonicum* have a low germination rate, which could restrict their survival and development^9,53^.

### Population sampling

The number of samples collected per population was in proportion to the size of that population. In order to avoid clonal interference for estimating genetic variation in populations, we collected only one sample per small patch (as in small patches all individuals may be ramets of the same genet); we did our best to collect at least 20–30% of all shoots per population. A total of 287 samples from 17 natural populations of *C*. *japonicum* were collected from China, Japan and Korea during four years (2010–2013), including small populations with only a few individuals (*n* = 3 in LS; Fig. 1 and Table 1). The collecting sites were chosen to approximately represent the complete distribution range of *C*. *japonicum*; the mean distance among sampled populations was 1,553.6 km, with a range of 44.2–3,834.5 km (in China, mean = 652.5 km, range = 44.2–1,443.5 km; in Japan, mean = 774.1 km, range = 175.3–1,279.1 km; the two Korean populations are separated by 74.2 km). We further divided the 11 Chinese populations into three groups according to geographic and physiographic criteria: DB, HS, LS and TM formed the “eastern” group, BT and SN the “central” group, and FP, JF, SZ, WX and ZP the “western” group. Population SZ, although geographically located in Hunan (province often included in central China according to Chinese geographers) was, instead, merged with the group of western populations because it occurs within Wuling Mts. (that actually constitute the eastern tip of the Yungui Plateau; Fig. 1). For each sampled individual, a small piece of fresh leaf was collected from a single plant and kept in a paper bag with silica gel until DNA extraction.

### DNA isolation

Total genomic DNA was extracted by using the modified CTAB method^66^ and dissolved in ultrapure water for future use. DNA quality was checked by electrophoresis in 1% agarose gels. Nanodrop was used to test its concentration and optical density value. For the ISSR experiment, working DNA was diluted to a concentration of 40–100 ng/μL in ultrapure water and stored at −20 °C before subsequent use. For the SCoT experiment, the most appropriate concentration of working DNA was 100–160 ng/μL. The materials used for ISSR analysis (*n* = 285) were slightly different from SCoT analysis (*n* = 265) due to technical constraints. In particular, samples of ZP could not be used for the SCoT analysis due to the degradation of materials (Table 1).

### ISSR and SCoT amplification and optimum primer selection

To ensure the reproducibility of both ISSR and SCoT markers, we first conducted many preliminary experiments to optimize PCR amplification (by trying several concentrations of DNA, primers, dNTPs, etc.), and then we chose primers with high polymorphism and good repeatability for subsequent manipulations. About 45% (ISSR) and 35% (SCoT) of the samples were re-run with all the primers, respectively. For ISSR analysis (*n* = 285), 10 primers were chosen from 100 ISSR primers supplied by University of British Columbia (http://www.biotech.ubc.ca/services/naps/primers.html) for PCR research (Supplementary Table S4). For SCoT analysis (*n* = 265), nine SCoT primers (Supplementary Table S5) were selected from 80 primers according to Collard and Mackill^30^ and Luo *et al*.^35^. Amplification with these primers was carried out in Takara TP600 and commenced with initial denaturation of 2 min at 94 °C, followed by 35 cycles of 35 s at 94 °C, 30 s annealing at 51–56.8 °C (SCoT: 51–53.4 °C), 90 s of elongation at 72 °C, and ending with a 7 min extension at 72 °C. The 15 μL reaction mixture contained 1 μL DNA, 7.5 μL 2 × Es Taq Master Mix (3 mM Mg^2+^, 0.4 mM dNTPs, Taq DNA polymerase and 2 × Es Taq PCR Buffer) (Biomiga, China), 40–100 ng DNA template and 0.9 μL (10 pmol/μL) primers. Amplification products were electrophoresed on a 2% agarose gel stained with 1% Goldview at 100 V for 30 min along with 100 bp DNA Ladder Marker, and photographed using the Bio-Rad Gel Documentation System. Reproducibly distinguishable bands were recorded and used in further analysis.

### Statistical analyses

Visible and clear DNA bands obtained from each ISSR and SCoT primers were scored as absent (0) or present (1). Bands length between 200–1,200 bp (ISSR) and 400–1,500 bp (SCoT) were scored. Using the program Popgene 1.32^67^, and assuming that populations were in Hardy–Weinberg equilibrium, we estimated the following genetic diversity parameters: percentage of polymorphic bands (*PPB*), observed number of alleles (*N*_a_), number of “private” alleles (*PA*), average effective number of alleles per locus (*N*_e_), Nei’s gene diversity index (*H*_E_)^68^, and Shannon’s information index (*SI*).

To assess the correspondence of genetic diversity between the two datasets (ISSR vs. SCoT), we performed Wilcoxon signed-rank tests on 16 population pairs (with the exception of population ZP) for five within-population genetic variation parameters. We further conducted a Spearman’s rank correlation analysis between ISSR and SCoT for each genetic measure; the higher correlation between ISSR and SCoT for each genetic measure, the smaller the differences in the measurements between the two datasets. Thus, this correlation analysis can be viewed as an indirect way to check the suitability of the SCoT markers in surveying the genetic variability in *C*. *japonicum*.

Although we found significant differences in the distribution of values of *H*_E_ and *SI* (Mann-Whitney *U-*test or Wilcoxon rank-sum test for both ISSR and SCoT, *P* = 0.000), the order of values of those two genetic parameters were nearly the same (Spearman’s rank correlation analysis for both ISSR and SCoT, *R*_S_^2^ = 0.995 and 0.985, respectively, *P* = 0.000), suggesting that using either *H*_E_ or *SI* would be appropriate. We used a Mann-Whitney *U-*test or Wilcoxon rank-sum test to assess the significance of differences in *H*_E_ (a summary statistic of within-population genetic variation) between the pair of regions with the highest and the lowest estimates in China. In addition, we further conducted Mann-Whitney *U-*test to determine any significant difference in *H*_E_ between the pair of countries with the highest and the lowest estimates in East Asia.

To estimate the degree of population genetic differentiation, we further calculated total genetic diversity (*H*_T_), genetic diversity within populations (*H*_S_), and genetic differentiation among populations (*G*_ST_) using Popgene. As the combination of dominant markers and small sample sizes could artificially inflate genetic structure (e.g., *G*_ST_), we estimated *G*_ST_ values with and without the four populations with small sample sizes [*n* < 10; LS (3 individuals), DB (5), Ha (7), and JF (8)] to see how much these small populations may be influencing our statistics. To test for the influence of individuals within populations, populations within regions (countries), and regions on the observed genetic variation, we conducted a hierarchical analysis of molecular variance (AMOVA) using GenAlEx 6.5^69^. To determine the relative importance of gene flow and RGD at regional scale^70^, we conducted a correlation analysis between pairwise genetic distances^71^ and linear geographic distances (km) and ran a Mantel test for IBD (with 999 permutations) using GenAlEx. A positively significant linear relation suggests that populations are at regional equilibrium between gene flow and RGD^70^. As reproductive barriers would exist between Chinese and Japanese populations due to different chromosome number (2*n* = 22 in China^60^; 2*n* = 20 in Japan^61,62^) and geographical isolation, we could be dealing with two cryptic species or subspecies. In order to avoid such potential “phylogenetic” biases in our genetic results, we performed further analyses of *G*_ST_ and IBD in a hierarchical fashion: within each country separately and then with all possible combinations of country pairs (i.e., “China vs. Korea”, “China vs. Japan”, and “Korea vs. Japan”).

We conducted unweighted pair-group method with arithmetic means (UPGMA) based on Nei’s unbiased genetic distances^72^ between populations using the Tfpga 1.3 program^73^. Bootstrap values for nodes were estimated based on 999 replications. As a complementary analysis, we performed a principal coordinate analysis (PCoA) with GenAlEx^69^, on the basis of Nei’s genetic distances^74^, to investigate the relationships among the populations, and the two principal coordinates were used to visualize the dispersion of accessions in a two-dimensional array of eigenvectors. To deeply explore the population structure and unravel genetic admixture, we used Structure 2.3.4^75^ to analyze the combined dataset (16 populations with ZP exclusion due to lack of SCoT data) of two markers with a Bayesian approach. Posterior probabilities of the data for each *K* were obtained for *K* = 1 to *K* = 18 clusters using the Admixture Model. Fifteen runs were completed for each *K*, with a Markov Chain Monte Carlo (MCMC) of 200,000 iterations, following a burn-in period of 1,000,000 iterations. We inferred the most likely value of *K* by the Δ*K* statistics^76^, with the aid of Structure Harvester^77^. Since the Δ*K* method tends to identify *K* = 2 as the top level of hierarchical structure^78^, we combined it with the method of selecting the smallest *K* after the log probability of data [ln Pr(*X|K*)] values reached a plateau^79^. Programs Clumpp 1.1.2^80^ and Distruct 1.1^81^ were used to combine the results of the 15 replicates of the best *K* and to visualize the results produced by Clumpp, respectively.

## Electronic supplementary material

Supplementary material
