# Supplementary material for: Genetic diversity in the endangered terrestrial orchid Cypripedium japonicum in East Asia: Insights into population history and implications for conservation
Source: Sci Rep. 2018 Apr 24;8:6467. doi: 10.1038/s41598-018-24912-z (PMC5915404; doi:10.1038/s41598-018-24912-z)
Supplement: Supplementary file 1 — Supplementary material [file 41598_2018_24912_MOESM1_ESM.doc]

**Genetic diversity in the endangered terrestrial orchid *Cypripedium japonicum* in East Asia: Insights into population history and implications for conservation**

**Huai Zhen Tian^1^, Li Xia Han^1^, Jun Li Zhang^1^, Xing Lin Li^1^, Takayuki Kawahara^2^, Tomohisa Yukawa^3^, Jordi López-Pujol^4^, Pankaj Kumar^5^, Myong Gi Chung^6^ & Mi Yoon Chung^7^**

^1^School of Life Sciences, East China Normal University, Shanghai 200241, China. ^2^Hokkaido Research Center, Forestry and Forest Products Research Institute, Sapporo, Hokkaido, Japan. ^3^Tsukuba Botanical Garden, National Museum of Nature and Science, Tsukuba, Ibaraki, Japan. ^4^Botanic Institute of Barcelona (IBB, CSIC-ICUB), Passeig del Migdia s/n, Barcelona 08038, Spain.^5^Kadoorie Farm & Botanic Garden, Lam Kam Rd., Lam Tsuen, Tai Po, New Territories, Hong Kong SAR, China. ^6^Department of Biology and the Research Institute of Natural Science (RINS), Gyeongsang National University, Jinju 52828, Republic of Korea. ^7^RINS, Gyeongsang National University, Jinju 52828, Republic of Korea. Correspondence and requests for materials should be addressed to H.Z.T. (email: [thz0102@126.com](mailto:thz0102@126.com)), M.G.C. (email: [mgchung@gnu.ac.kr](mailto:mgchung@gnu.ac.kr)) or M.Y.C. (email: [miyoon71@gnu.ac.kr](mailto:miyoon71@gnu.ac.kr))

Supplementary Fig. S1. Relationship between genetic and geographic distance based on ISSR (A: *r* = 0.713 *P* = 0.001) and SCoT (B: *r* = 0.515, *P* = 0.001).


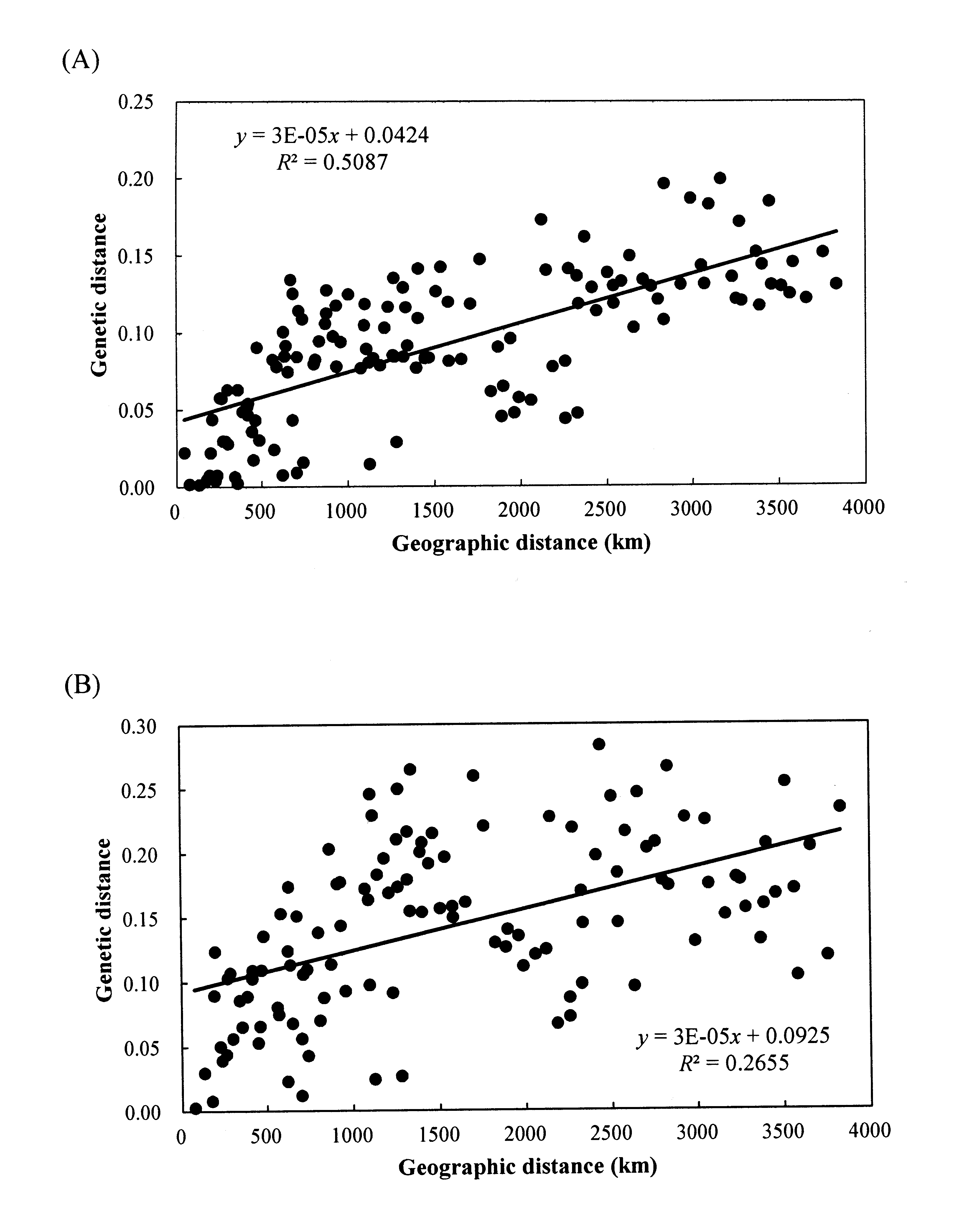


Supplementary Table S1. Summary of Wilcoxon signed-rank tests and Spearman’s rank correlation analysis between ISSR and SCoT for each genetic measure.

| Parameter |  | Wilcoxon signed-rank test | |  | ISSR vs. SCoT correlation | |
| --- | --- | --- | --- | --- | --- | --- |
|  |  | *Z* | *P* |  | *R*^2^ | *P* |
| *PPB* |  | -3.095 | 0.002 |  | 0.930 | < 0.000 |
| *N*_a_ |  | -2.446 | 0.014 |  | 0.930 | < 0.000 |
| *PA* |  | -0.311 | 0.756 |  | 0.107 | 0.217 |
| *N*_e_ |  | -2.578 | 0.010 |  | 0.645 | 0.000 |
| *H*_E_ |  | -2.508 | 0.012 |  | 0.791 | < 0.000 |
| *SI* |  | -2.783 | 0.005 |  | 0.863 | < 0.000 |

*Notes:* *R*_S_^2^, coefficient of determination.

Supplementary Table S2. Hierarchical analysis of *G*_ST_ and correlation between pairwise genetic distances and linear geographic distances (km) of populations (test for isolation by distance, IBD) for all populations, within each country, and with all possible combinations of country pairs, of *Cypripedium japonicum* based on ISSR/SCoT markers.

| Source of variation | *G*_ST_ | |  | *R*^2^ (*P*) | |
| --- | --- | --- | --- | --- | --- |
|  | ISSR | SCoT |  | ISSR | SCoT |
| All populations | 0.749 | 0.779 |  | 0.5087 (0.001) | 0.2655 (0.001) |
| Populations within China | 0.636 | 0.697 |  | 0.5661 (0.004) | 0.4480 (0.003) |
| Populations within Korea | 0.104 | 0.145 |  | - | - |
| Populations within Japan | 0.321 | 0.406 |  | 0.7369 (0.036) | 0.2288 (0.262) |
| China versus Korea^1^ | 0.120 | 0.159 |  | 0.1412 (0.007) | 0.1310 (0.010) |
| China versus Japan^1^ | 0.335 | 0.300 |  | 0.6120 (0.001) | 0.3603 (0.001) |
| Korea versus Japan^1^ | 0.672 | 0.608 |  | 0.4724 (0.007) | 0.5583 (0.015) |

*Notes: G*_ST_*,* genetic differentiation coefficient; *R*^2^, coefficient of determination.

^1^For the calculations of *G*_ST_ with all possible combinations of country pairs, all populations within each country were merged, so *G*_ST_ was estimated between two groups of populations; for the tests of IBD, in contrast, populations within each country were not merged.

Supplementary Table S3. Levels and distribution of genetic diversity within and among populations of orchid species examined by ISSR markers. This table is modified and updated from Table 4 in Qian *et al*. (2014).

| Taxon | *PPB*_S_ (%) | *PPB*_P_ (%) | *H*_ES_ | *H*_EP_ | *SI*_S_ | *SI*_P_ | *G*_ST_ (*F*_ST_) | Reference |
| --- | --- | --- | --- | --- | --- | --- | --- | --- |
| *Amitostigma hemipilioides* | 64.7 | 50.9 | 0.686 | 0.603 | 0.387 | 0.295 | 0.367 | Yang *et al*. (2014) |
| *Brassavola tuberculata* | 52.4 | na | 0.216 | na | 0.314 | na | na | Fajardo *et al.* (2014) |
| *Calanthe tsoongiana* | 96.8 | 50.0 | 0.398 | 0.183 | 0.576 | 0.271 | 0.550 | Qian *et al.* (2013) |
| *Cattleya bicolor* | 56.6 | na | 0.219 | na | 0.323 | na | na | Fajardo *et al.* (2014) |
| *C. elongata* | na | 56.8 | na | na | na | na | 0.180 | Da Cruz *et al.* (2011) |
| *C. granulosa* | 30.7 | na | 0.163 | na | 0.273 | na | na | Fajardo *et al.* (2014) |
| *C. labiata* | 30.1 | na | 0.132 | na | 0.193 | na | na | Fajardo *et al.* (2014) |
| *C. schofieldiana* | 56.0 | na | 0.213 | na | 0.314 | na | na | Fajardo *et al.* (2014) |
| *Cymbidium goeringii* | 88.2 | 63.1 | 0.263 | 0.195 | 0.404 | 0.296 | 0.244 | Yao *et al.* (2007) |
| *C. kanran* | 80.5 | 68.3 | 0.303 | 0.224 | 0.389 | 0.351 | na | Kim *et al.* (2015) |
| *Cypripedium japonicum* | 38.5 | 11.2 | 0.127 | 0.042 | 0.193 | 0.061 | 0.671 | Qian *et al.* (2014) |
| ***C. japonicum*** | **57.7** | **9.7** | **0.104** | **0.027** | **0.169** | **0.042** | **0.749** | **This study** |
| *Dendrobium fimbriatum* | 89.7 | 23.9 | 0.323 | 0.087 | 0.478 | 0.129 | 0.744 | Ma & Yin (2009) |
| *Gastrodia elata* | 81.8 | 59.1 | 0.236 | 0.176 | 0.367 | 0.270 | 0.273 | Wu *et al.* (2006) |
| *Octomeria crassifolia* | na | 91.6 | 0.352 | 0.265 | 0.530 | 0.401 | 0.760 | Barbosa *et al.* (2013) |
| *O. grandiflora* | na | 82.4 | 0.338 | 0.258 | 0.508 | 0.382 | 0.120 | Barbosa *et al.* (2013) |
| *Paphiopedilum micranthum* | 91.7 | 80.3 | 0.384 | 0.285 | 0.565 | 0.424 | 0.258 | Huang *et al.* (2014) |
| *Piperia yadonii* (2006) | na | na | na | 0.062 | na | na | 0.424 | George *et al.* (2009) |
| *P. yadonii* (2007) | na | na | na | 0.059 | na | na | 0.394 | George *et al.* (2009) |
| *Platanthera aquilonis* | 61.7 | 22.6 | 0.184 | 0.084 | na | na | 0.700 | Wallace (2004) |
| *P. dilatata* | 57.5 | 35.4 | 0.182 | 0.131 | na | na | 0.490 | Wallace (2004) |
| *P. huronensis* | 43.0 | 32.6 | 0.172 | 0.119 | na | na | 0.360 | Wallace (2004) |
| *Tipularia discolor* | na | 8.0 | na | 0.031 | na | na | 0.415 | Smith *et al.* (2002) |
| **Average** | **63.4** | **46.6** | **0.263** | **0.167** | **0.374** | **0.266** | **0.453** |  |

*Notes:* The subscript “S” denotes species’ (or pooled samples) values, while the subscript “P” indicates population means. na, not available.

**References**

Barbosa, A. R., Silva-Pereira, V. & Borba, E. L. High genetic variability in self-incompatible myophilous *Octomeria* (Orchidaceae, Pleurothallidinae) species. *Braz. J. Bot.* **36,** 179–187 (2013).

Da Cruz, D. T., Selbach-Schnadelbach, A., Lambert, S. M., Ribeiro, P. L. & Borba, E. L. Genetic and morphological variability in *Cattleya elongata* Barb. Rodr. (Orchidaceae), endemic to the campo rupestre vegetation in northeastern Brazil. *Plant Syst. Evol.* **294,** 87–98 (2011).

Fajardo, C. G., De Almeida Vieira, F. & Molina, W. F. Interspecific genetic analysis of orchids in Brazil using molecular markers. *Plant Syst. Evol.* **300,** 1825–1832 (2014).

George, S., Sharma, J. & Yadon, V. L. Genetic diversity of the endangered and narrow endemic *Piperia yadonii* (Orchidaceae) assessed with ISSR polymorphisms. *Am. J. Bot.* **96,** 2022–2030 (2009).

Huang, J. L., Li, S. Y. & Hu, H. ISSR and SRAP markers reveal genetic diversity and population structure of an endangered slipper orchid, *Paphiopedilum micranthum* (Orchidaceae). *Plant Divers. Resour.* **36,** 209–218 (2014).

Kim, E. H. *et al*. Genetic diversity and spatial structure of a population the natural monument (no. 432) *Cymbidium kanran* in Sanghyo-dong, Jeju-do. *J. Agric. Life. Sci.* **49,** 1–11 (2015).

Ma, J. M. & Yin, S. H. Genetic diversity of *Dendrobium fimbriatum* (Orchidaceae), an endangered species, detected by inter-simple sequence repeat (ISSR). *Acta Bot. Yunnanica*. **31,** 35–41 (2009).

Qian, X., Wang, C. & Tian, M. Genetic diversity and population differentiation of *Calanthe tsoongiana*, a rare and endemic orchid in China. *Int. J. Mol. Sci.* **14,** 20399–20413 (2013).

Qian, X. *et al.* Conservation genetics of an endangered lady’s slipper orchid: *Cypripedium japonicum* in China. *Int. J. Mol. Sci.* **15,** 11578–11596 (2014).

Smith, J. L., Hunter, K. L. & Hunter, R. B. Genetic variation in the terrestrial orchid *Tipularia discolor*. *Southeast Nat.* **1,** 17–26 (2002).

Wallace, L. E. A comparison of genetic variation and structure in the allopolyploid

*Platanthera huronensis* and its diploid progenitors, *Platanthera aquilonis* and *Platanthera dilatata*. *Can. J. Bot.* **82,** 244–252 (2004).

Wu, H. F., Li, Z. Z. & Huang, H. W. Genetic differentiation among natural populations of *Gastrodia elata* (Orchidaceae) in Hubei and germplasm assessment of the cultivated populations. *Biodivers. Sci.* **14,** 315–326 (2006).

Yang, Q. *et al*. Genetic diversity and differentiation in the critically endangered orchid (*Amitostigma hemipilioides*): Implications for conservation. *Plant Syst. Evol.* **300,** 871–879 (2014).

Yao, X. H., Gao, L. & Yang, B. Genetic diversity of wild *Cymbidium goeringii* (Orchidaceae) populations from Hubei based on inter-simple sequence repeats analysis. *Frontiers Biol. China*. **2,** 419–424 (2007).

Supplementary Table S4. Primers used for ISSR-PCR and number of bands per primer.

| Primers |  | Sequence (5’-3’) | Annealing temperature | No. of amplified bands | No. of polymorphic bands |
| --- | --- | --- | --- | --- | --- |
| UBC810 |  | (GA)8T | 51.0ºC | 9 | 1 |
| UBC811 |  | (GA)8C | 52.0ºC | 6 | 3 |
| UBC818 |  | (CA)8G | 51.0ºC | 6 | 2 |
| UBC825 |  | (AC)8T | 52.0ºC | 7 | 5 |
| UBC835 |  | (AG)8YC | 54.4ºC | 8 | 4 |
| UBC836 |  | (AG)8YA | 54.0ºC | 7 | 5 |
| UBC840 |  | (GA)8YT | 54.0ºC | 6 | 3 |
| UBC842 |  | (GA)8YG | 56.8ºC | 8 | 5 |
| UBC857 |  | (AC)8YG | 52.0ºC | 14 | 10 |
| UBC866 |  | (CTC)6 | 55.6ºC | 7 | 4 |

Y = (C,T)

Supplementary Table S5. Primers used for SCoT-PCR and number of bands per primer.

| Primers | Sequence (5’-3’) | Annealing | No. of | No. of |
| --- | --- | --- | --- | --- |
|  |  | temperature | amplified bands | polymorphic bands |
| SCoT16 | ACCATGGCTACCACCGAC | 51.0ºC | 10 | 7 |
| SCoT17 | ACCATGGCTACCACCGAG | 51.0ºC | 11 | 5 |
| SCoT35 | CATGGCTACCACCGGCCC | 52.5ºC | 6 | 4 |
| SCoT37 | CAATGGCTACCACTAGCC | 51.5ºC | 9 | 6 |
| SCoT43 | CAATGGCTACCACCGCAG | 51.0ºC | 9 | 2 |
| SCoT66 | ACCATGGCTACCAGCGAG | 53.4ºC | 8 | 5 |
| SCoT73 | CCATGGCTACCACCGGCT | 51.0ºC | 10 | 7 |
| SCoT75 | CCATGGCTACCACCGGAG | 51.0ºC | 8 | 6 |
| SCoT78 | CCATGGCTACCACTAGCA | 51.0ºC | 6 | 5 |
